# Recent Advances in ROS-Responsive Cell Sheet Techniques for Tissue Engineering

**DOI:** 10.3390/ijms20225656

**Published:** 2019-11-12

**Authors:** Min-Ah Koo, Mi Hee Lee, Jong-Chul Park

**Affiliations:** 1 Department of Medical Engineering, Yonsei University College of Medicine, Seoul 03722, Korea; 2 Brain Korea 21 PLUS Project for Medical Science, Yonsei University College of Medicine, Seoul 03722, Korea

**Keywords:** cell sheet, cell detachment, reactive oxygen species

## Abstract

Cell sheet engineering has evolved rapidly in recent years as a new approach for cell-based therapy. Cell sheet harvest technology is important for producing viable, transplantable cell sheets and applying them to tissue engineering. To date, most cell sheet studies use thermo-responsive systems to detach cell sheets. However, other approaches have been reported. This review provides the progress in cell sheet detachment techniques, particularly reactive oxygen species (ROS)-responsive strategies. Therefore, we present a comprehensive introduction to ROS, their application in regenerative medicine, and considerations on how to use ROS in cell detachment. The review also discusses current limitations and challenges for clarifying the mechanism of the ROS-responsive cell sheet detachment.

## 1. Introduction

The development of tissue engineering technology has shown considerable potential in the field of regenerative medicine [1]. Tissue engineering is based on conventional methods of seeding cells into biodegradable scaffolds that replace the extracellular matrix (ECM). It has already demonstrated clinical results that can be transplanted into various animal models to restore or enhance original tissue functions [2,3,4,5]. These results indicated the emergence of better regenerative therapies than cell suspension injection.

A “cell sheet engineering” strategy of harvesting cultured cells as intact sheets with deposited ECM has been developed using temperature-responsive surfaces and can be applied in tissue engineering. The temperature-responsive surfaces allow harvesting cultured cells without the use of proteolytic enzymes, such as trypsin or dispase, which can lead to cell membrane and surface antigen damage and loss of differentiated phenotypes [6,7]. In addition, cell sheets with ECM can be transplanted directly to the target site or stacked to produce structures similar to three-dimensional (3D) tissue [8]. This approach may address the limitations of existing regenerative therapies, such as cell injection and tissue reconstruction using biodegradable scaffolds. Single cell suspension injection results in significant loss of cells because the cells are not grafted at the target site, and the use of scaffolds also results in cell damage due to insufficient supply of oxygen and nutrients to the cells. On the other hand, cell sheets preserved with ECM can adhere tightly to host tissues or lesions and minimize cell loss [2].

Cell sheet harvest technology have been developed to obtain viable, transplantable cell sheets for various applications in tissue engineering. Most cell sheet studies use temperature-responsive systems for cell sheet detachment. However, various systems that respond to different stimuli also show the potential for cell sheet harvesting. This review provides an overview of current techniques for creating cell sheets using various systems, particularly reaction oxygen species (ROS)-responsive systems.

## 2. Cell Sheet Harvesting Methods

### 2.1. Thermo-Responsive Systems

**Figure 1:**
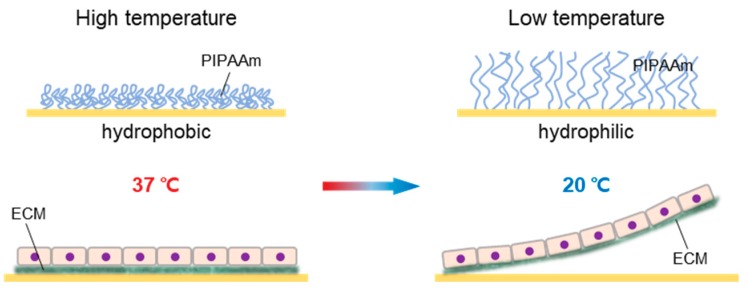
Schematic illustrations of cell sheet detachment from PIPAAm-grafted culture surfaces. The PIPAAm converts the wettability of the surface from hydrophobic to hydrophilic as the temperature changes. Cells attach to hydrophobic culture surface (37 °C) via ECM, and are linked to each other via cell-to-cell junction proteins. Cells are cultured on thermo-responsive culture surfaces, and the attachment between ECM and the hydrophilic culture surface is released only by low temperature at ˂32 °C.

The most widely studied thermo-responsive system is based on a temperature-responsive culture dish that is covalently grafted with poly(N-isopropylacrylamide) (PIPAAm) [9,10,11,12]. This polymer shows a distinct transition from hydrophobic to hydrophilic states at its lower critical solution temperature (LCST) of 32 °C [13]. Cells can adhere and proliferate on the culture dish because the PIPAAm is hydrophobic at the conventional culture temperature (37 °C), but when the temperature decreases to 32 °C, the cells cannot attach to the culture dish due to rapid hydration and swelling of the grafted PIPAAm [13,14,15,16]. Consequently, cells that form a layer on the surface at 37 °C can be harvested as an intact cell sheet by simply reducing temperature below the LCST of PIPAAm polymer within 1 h (Figure 1) [17,18]. This method is currently being applied to research harvest varieties of cell sheets.

However, the use of PIPAAm alone may affect cellular metabolic function due to low temperatures, since it takes more than 30 min at 20 °C to harvest cell sheets [19,20]. Several methods have been developed to fabricate PIPAAm-modified thermo-responsive surfaces to reduce cell detachment time. Above all, electron beam (EB) polymerization is the most widely used method to graft PIPAAm onto tissue culture polystyrene (TCPS), but cells must be maintained at 20 °C for 1 h [15,21,22,23]. Similarly to EB irradiation, plasma and ultraviolet (UV) irradiation are used as alternative approaches for producing a PIPAAm-grafted surface. With the plasma irradiation method, bovine aortic endothelial cell (BAEC) sheets were harvested from the surface by decreasing the temperature at 20 °C for 2 h [24,25,26]. The other way to graft PIPAAm is through UV irradiation, which reduces the detachment time from 1–2 h to 30 min [27,28]. Solvent casting methods were also used to produce bulk PIPAAm sheets. Instead of grafting onto a substrate, the PIPAAm sheets are conjugated with collagen and deposited on a solid support, such as TCPS or glass, to reduce the detachment time to 20 min [20,29]. Another new approach is spin-coating techniques, which deposit PIPAAm thin films on substrates without the expensive equipment used for EB or plasma polymerization. Nash et al. [30] spin-coated PIPAAm/ethanol mixtures to fabricate a thin film, which showed rapid cell sheet detachment in various cell types within 5–10 min (in some cases up to 60 min) by treatment with 4 °C media. However, poor cell growth and adhesion on spin-coated PIPAAm films have resulted in the detachment of some clumps rather than entire cell sheets [31]. To this end, Patel et al. developed thermo-responsive films in which PIPAAm blended with 3-aminopropyltriethoxysilane (APTES) was deposited on glass slides to provide anchor points for cell attachment and proliferation on the film surface produced by the spin-coating technique [32]. By changing the PIPAAm to APTES ratio, this method can control the cell sheet detachment time ranging from 2.5 to 40 min. This spin coating technique is simple and economical for harvesting cell sheets, but only applied studies using commercially available thermo-responsive surfaces (UpCell^®^) made by EB polymerization have been reported. Recently, a method using thermosensitive Tetronic^®^-based hydrogels has been reported, which can detach various cell sheets at the same time as the size expansion caused by dropping the temperature below 37 °C. This method took less than 10 min at 4 °C or more than 15 min at 25 °C [33,34].

### 2.2. Electro-Responsive Systems

In the electro-responsive system developed by the Mrksich group [35], electroactive self-assembled monolayers (SAMs) on gold are used to immobilize ligands. The electroactive molecules are tethered to the monolayer oxidize when electrical potential is applied to the gold film where the immobilized ligands are released. The system can be electrically converted to use cell adhesion-mediating peptide ligands to trigger cell attachment or detachment. Fukuda et al. [36] used a similar electrical responsive system for efficient cell sheet detachment. Their design took the form of gold-thiolate bonds on gold-coated substrates with the tripeptide Arg-Gly-Asp (RGD)-containing oligopeptides. Fibroblast sheets were then detached within 10 min of applying −1.0 V electrical potential to the surface. Cell sheet detachment was caused by the peptide release from the gold substrate by electrical stimulation. Another electro-responsive system that induces cell sheet detachment involves the use of polyelectrolyte-modified surfaces [37]. Polyelectrolytes adsorbs on the oppositely charged surface due to the electrostatic interaction and desorbs from the conductive substrate during electrochemical polarization. Through this mechanism, the cell sheet detaches from the surface together with polyelectrolytes [38,39]. However, using polyelectrolyte-modified surfaces, local pH changes due to electrochemical dissolution of polyelectrolyte coatings can cause DNA damage and apoptosis [31,40].

### 2.3. pH-Responsive Systems

Although pH-responsive systems are difficult to apply in cell-based applications due to the limited range of pH (6.8–7.4) for normal cell function, Ehrbar et al. [41] reported that cell sheet detachment can be controlled by local or global pH drop. A current density of 30 µA/cm^2^ applied to the pH-responsive substrates fabricated by alternating stacks of cationic poly(allylamine hydrochloride) layers and anionic poly(styrene sulfonate) layers on a conductive indium tin oxide surface detached the cells with intact ECM within 10–20 min [41]. In addition, the Ehrbar group hypothesized that the reduction in local pH at the cell–substrate interface leads to cell sheet detachment. Thus, instead of using an electrical trigger to detach cell sheets, cells were allowed to detach by decreasing bulk pH through change in culture media pH [31]. The range of pH 5.0 to 7.4 did not show a change in cell adhesion, whereas pH 4.0 resulted in complete cell sheet detachment within 2–3 min. Thus, the approach with the pH responsive substrate used in this study showed an alternative method of releasing cell sheets from the surface, although damage of cells sensitive to pH change could not be avoided.

### 2.4. Magnetic Systems

Ito et al. developed magnetite cationic liposomes (MCLs) [42], which are cationic liposomes containing magnetite nanoparticles, in order to improve accumulation of magnetite nanoparticles in target cells. This study confirmed that placing a magnet under ultralow-attachment plates with a surface consisting of a covalently bonded hydrogel layer that is hydrophilic and neutrally charged, incubating keratinocytes, and then, removing the magnet, could harvest the cells from the plate without enzymatic treatment. To detach the cell sheet, the magnet was removed from under the culture plate and a polyvinylidene fluoride (PVDF) membrane was placed on the surface of the magnet. The magnet was moved to the top of the cells, and the keratinocyte sheets stuck to the PVDF membrane on the magnet surface. The PVDF membranes were used to transfer cell sheets detached from magnets to new locations. This magnetic system has been applied to detach and transfer cell sheets of various cell types, including keratinocytes, cardiomyocytes, hepatocytes, endothelial cells, mesenchymal stem cells, and retinal pigment epithelial cells [42,43,44,45,46]. Although the use of magnetite nanoparticles has not been reported to cause cytotoxicity, the disadvantage is that pure cell sheets cannot be obtained. In addition, the system is effective in releasing and transferring cells, but the cell sheets do not detach into cell monolayers and form aggregates in which the cells clump.

## 3. ROS-Responsive Methods

This section introduces methods of detaching cell sheets by ROS and discusses the effects of ROS on cell detachment.

### 3.1. Definition of ROS

ROS are natural byproducts of cellular oxidative metabolism and are involved in the regulation of cell survival, cell death, differentiation, cell signaling, and inflammation-related factor production [47,48]. Biologically important ROS elements include free radicals, such as singlet oxygen (^1^O_2_), superoxide (O_2_^•–^), hydroxyl (HO^•^), hydroperoxyl (HO_2_^•^), carbonate (CO_3_^•–^), peroxyl (RO_2_^•^), alkoxyl (RO^•^), and carbon dioxide radicals (CO_2_^•–^), and nonradicals, such as hydrogen peroxide (H_2_O_2_), hypobromous acid (HOBr), hypochlorous acid (HOCl), ozone (O_3_), organic peroxides (ROOH), peroxynitrite (ONOO^–^), peroxynitrate (O_2_NOO^–^), peroxynitrous acid (ONOOH), peroxomonocarbonate (HOOCO_2_^–^), nitric oxide (NO), and hypochlorite (OCl^–^) [49,50,51]. Originally, only phagocytic cells were known to be responsible for ROS production in host cell defense mechanisms. Recent studies have shown that ROS play a role in cell signaling, including apoptosis, gene expression, and the activation of cell signaling cascades [52]. In particular, ROS acts differently in cells depending on the concentration. Low levels of ROS activate cell signaling pathways to initiate biological processes [53]. However, high levels of ROS cause cellular and DNA damage, and activation of cell death processes, such as apoptosis, depending on the severity and duration of exposure.

### 3.2. Source of ROS Generation

**Figure 2:**
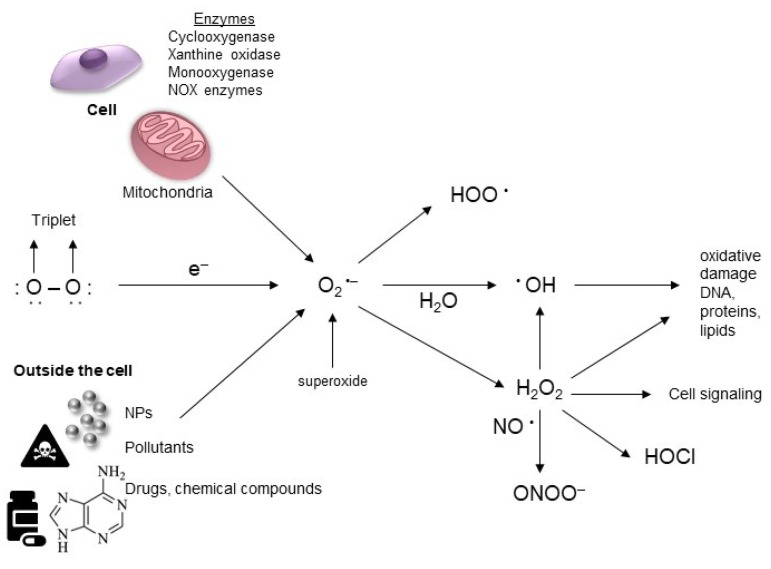
Mechanisms for the generation of intracellular and extracellular ROS (reactive oxygen species). Extracellular ROS are generated from environmental pollutants, drugs, xenobiotic substances, or radiation. Intracellular ROS are known to be generated through multiple mechanisms inside the cell (metabolic by-products of biological systems).

The main sources of intracellular ROS are mitochondria, the endoplasmic reticulum (ER), peroxisomes, microsomes, and nicotinamide adenine dinucleotide phosphate (NADPH) oxidase (NOX) complexes in cell membranes [49,54,55]. In particular, mitochondria are the main intrinsic source of ROS production via the mitochondrial electron-transport system [56]. Extracellular sources also contribute to ROS generation, such as radiation, pollutants, nanoparticles (NPs), and various drugs, and certain types of other chemical compounds also play a role [57,58,59]. NPs of metals can induce the generation of the radical reactive superoxide by donating an electron to molecular oxygen; the superoxide then triggers a cascade of radical forming reactions [60], as shown in Figure 2.

**Figure 3:**
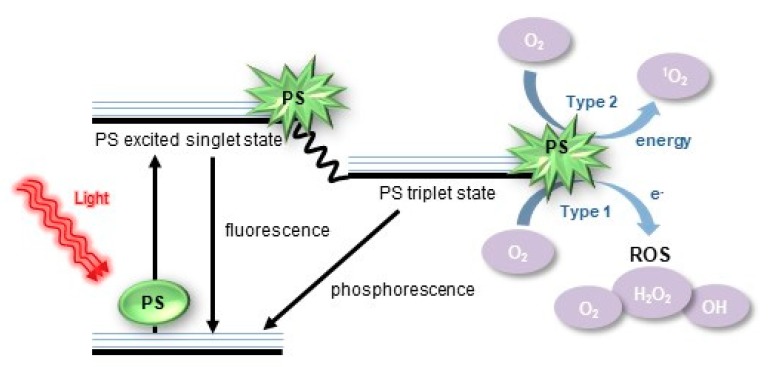
Schematic illustrations of the process of generating extracellular ROS from a photosensitizer under light irradiation. PS: photosensitizer.

Photodynamic action (PDA) is a method of inducing the generation of ROS. Integral to PDA are a photosensitizer (PS), a light-absorbing molecule, and a light source with a suitable wavelength. With light irradiation, the PS absorbs the light energy and transfers to an excited state. The excited PS then undergoes a photochemical reaction (PR) with a biological environment to generate ROS, which is called PDA [61]. There are two main types of PDA: type I reaction involves electron transfer PR to generate radical and radical anion species, while type II reaction directs PR through energy transfer between oxygen and excited PS, to produce singlet oxygen (Figure 3) [62,63,64,65].

### 3.3. Cell Harvesting Methods by Extracellular ROS

**Figure 4:**
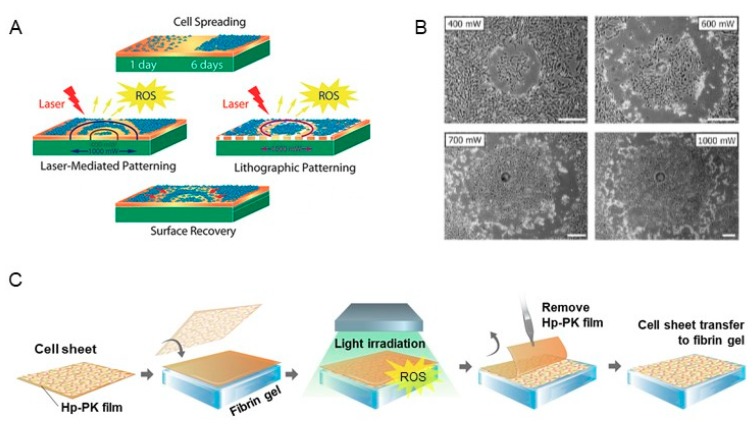
(**A**) Schematics of cell detachment by laser irradiation on AuNP-based surfaces. (**B**) Phase contrast microscopic images showing cell detachment areas according to the laser power. The images were taken 24 h after irradiation (Reprinted with permission from [68]. Copyright (2019) American Chemical Society). (**C**) Schematic illustration of the ROS-induced cell sheet detachment and transfer procedure on Hp-PK films. (Reprinted from [69] with permission of Elsevier and Copyright Clearance Center).

Studies have reported that ROS act as mediators of cell adhesion [66], and an increase in intracellular ROS levels can lead to cell detachment [67]. The Möhwald group [68] reported the use of light to release fibroblasts cultured on gold nanoparticle-based surfaces. Gold nanoparticles (AuNP) have strong light absorption in the green spectral range. Thus, the authors irradiated the surface with a green laser (532 nm) to produce extracellular ROS by a photochemical mechanism (Figure 4A). The ROS damaged cell membranes at the cell–surface interface, detaching the cells from the substrate. The cells did not immediately detach from the surface, but took up to 24 h to completely detach. One of the advantages of this system is that the surface can be recovered, allowing the cells to reattach in the irradiated areas within 72 h. This property is able to spatially pattern cells, and control the area where the green laser is irradiated to produce co-cultured cell sheets that can be reattached by seeding different cell types after the surface has recovered (Figure 4B). This method has been reported to be applicable to the individual detachment of cells from the culture surface, but has not shown the results of detaching the cells in sheet form.

Recently, we reported a new ROS-induced strategy for direct transfer of intact cell sheets to target sites without intermediate harvesting processes based on hematoporphyrin-incorporated polyketone (Hp-PK) film [69]. After green light emitting diode (LED) (510 nm) irradiation for 5–10 min, exogenous ROS generated from the Hp-PK film induced cell sheet detachment and transfer simultaneously. Briefly, this process is carried out by placing a cell cultured film on the target site, irradiating light, and then peeling only the film (Figure 4C). We have successfully applied the detachment of various cell types in a simple way to control the irradiation power and time. In addition, our strategy was applied to the in vivo transplantation onto subcutaneous tissue. As mentioned above, cell sheets can adhere to tissues because of the presence of ECM. Therefore, we stacked the cell sheets in multiple layers and transplanted them by the ROS-induced method, and confirmed that the stacked cell sheets were well grafted at the transplant site without cell loss. In particular, multi-layered stem cell sheets accelerated wound healing in full-thickness skin defects [70]. The currently reported single cells or cell sheet detachment methods have inevitably resulted in cell damage since the cells have to be left at a low temperature or low pH environment for a long time [19,20,40]. In addition, the ligands and magnetic NPs, which are used for cell detachment, are released together with the cells. Thus, there is a limit in harvesting only an intact cell sheet. Our system addresses these limitations and demonstrates the possibility of efficient cell transplantation into the lesion site for tissue regeneration and reconstruction, the ultimate goal of cell sheet engineering.

The advantage of the methods using extracellular ROS is the ease of spatio-temporal control of cell detachment. ROS have a short half-life (<40 ns) and can only act close to the site of generation (<20 nm), which is significantly less than cell dimensions [71,72]. Thus, on a molecular scale, ROS can only act chemically at a small distance from the site of their production. Due to this property, the Möhwald group's research [68] mentioned above shows that spatial control is possible by ensuring that the cells attached to the surface of AuNP undergo patterned detachment according to the ring-like profile of the laser beam. In addition, the PS-incorporated film used in our study [69] suggested that the occurrence of unintentional cell detachment can be avoided by spatially controlling the generation of ROS and controlling the production time with or without light stimulation.

### 3.4. Current Limitation for Clarifying the Mechanism by ROS

Because cell sheet detachment by extracellular ROS occurs gradually, some studies have reported that this change is due to cell signaling initiated by ROS [73,74]. The downstream effect of ROS production is the generally reversible oxidation of proteins [66,75]. Redox-sensitive proteins, which include protein tyrosine phosphatases (PTPs) as the active site cysteine, are the target of specific oxidation by various oxidants [76]. Focal adhesion kinase (FAK) is a non-receptor protein tyrosine kinase that plays an important role in signal transduction from integrin-enriched focal adhesion (FA) sites that mediate cellular contact with the extracellular matrix. Multiple protein–protein interaction sites of FAK mediate association with adapters and structural proteins [77,78]. Chiarugi et al. [66] provide evidence that ROS take a role in integrin signaling. In the role for oxidative species in integrin signaling, ROS generated during cell adhesion induce up-regulation of FAK. However, increasing of intracellular ROS up to a threshold level delays cell adhesion to ECM proteins, and results in a negative function of PTP on FA development and cytoskeleton organization. We previously found that extracellular ROS increase the amount of intracellular ROS [79]. H_2_O_2_ can diffuse through specific aquaporins (AQP) in the plasma membrane, and superoxide anion (O_2_^–^) can penetrate the cell membrane through anion channels (Cl^–^ channel-3) to initiate intracellular signal transduction [79,80,81,82]. There are many ways in which extracellular ROS can be transported into cells, but the exact mechanism of ROS that affects cell detachment is unknown. In contrast, our study using FTIR spectroscopy demonstrates that ROS-induced cell detachment is due to secondary structural changes in proteins adsorbed on the Hp-PK films [69]. Thus, we expect that these conformational changes are caused by extracellular ROS in ECM proteins present in the cell sheet. However, the type of ROS that affects cell detachment has not been identified.

ROS are difficult to distinguish and quantify from each element by specific assays. These other reactive molecules have properties of overlapping or distinguishing from each other. Some scientists have reported that various types of ROS can be distinguished by specific probes and categorized several probes for each ROS (Table 1) [83,84,85,86]. There are various ROS probes that can be analyzed by flow cytometry or microscopy [87,88]. However, most of these are not specific to a particular ROS species, are unstable, and can be affected by other factors distinct from the oxidants. Therefore, when using these probes, it is necessary to carefully interpret the data derived by comparing the various methods.

## 4. Final Remarks and Outlook

Various stimuli-induced methods have been developed to enable the detachment of cell sheets, but more research is needed to reach the level of direct cell transplantation, the most important step in tissue engineering. Therefore, one of the major challenges to be overcome in the future is to develop simple and economical methods that can not only detach cell sheets but also transplant them. Detaching cell sheets using the most commonly used thermo-responsive surfaces can affect cell function because the cells are exposed to low temperatures for more than 30 min. Several of the techniques discussed in this review article try to improve cell detachment time. However, these techniques have limitations in practice for use in cell transplantation. ROS-responsive methods have the advantage of being capable of space–time control. In particular, using a PS-incorporated polymer film, cell sheet detachment and transplantation can be performed in one step compared to conventional methods. This is a significant advantage when creating 3D tissue constructs or applying them to regenerative therapies. Although it is difficult to identify the types and mechanisms of exogenous ROS that induce cell detachment, cell sheet engineering can be actively applied to clinical treatment and fabrication of biomimetic tissue with further improvement, such as the efficiency of ROS generation.

## Figures and Tables

**Table 1:** Methods for the detection of ROS. (Reproduced from [86] by permission of The Royal Society of Chemistry).

Probe	Specificity	Advantages	Disadvantages
Fluorescent probes	O2^•^^―^, H_2_O_2_	Cell permeable, intensity quantifiable, product stable	Products complex, low specificity, interfered by ONOO^―^
Chemiluminescent probes	O2^•^^―^, ·OH	Cell permeable	Low selectivity and sensitivity, intermediates not stable
Spectrophotometry methods	O2^•^^―^, H_2_O_2_	Sensitive, fast, single product	Low specificity
Chromatography methods	·OH	Fast, sensitive	Products complex
Electrochemical biosensors	O2^•^^―^	Sensitive, fast	Complex to prepare
Electron spin resonance	ROS, RNS	Specific, sensitive	Expensive
Fluorescent proteins	H_2_O_2_, redox status changes	Dynamic, real-time, cell friendly	Slow in reaction, restriction in receptor cells, non-sensitive
